# Demonstration of VOC Fenceline Sensors and Canister Grab Sampling near Chemical Facilities in Louisville, Kentucky

**DOI:** 10.3390/s22093480

**Published:** 2022-05-03

**Authors:** Megan MacDonald, Eben Thoma, Ingrid George, Rachelle Duvall

**Affiliations:** 1 Oak Ridge Institute of Science and Engineering Fellowship Program, Oak Ridge, TN 37830, USA; macdonald.megan@epa.gov; 2 U.S. Environmental Protection Agency, Office of Research and Development, Center for Environmental Measurement and Modeling, Research Triangle Park, Durham, NC 27709, USA; george.ingrid@epa.gov (I.G.); duvall.rachelle@epa.gov (R.D.)

**Keywords:** fenceline monitoring, facility emissions, 1,3-butadiene, cyclohexane, SPod, sensor

## Abstract

Experimental fenceline sensor pods (SPods) fitted with 30 s duration canister grab sampling (CGS) systems were deployed at a site near chemical facilities in Louisville, KY, from 4 June 2018 to 5 January 2020. The objective of the study was to better understand lower cost 10.6 eV photoionization detector (PID)-based volatile organic compound (VOC) sensors and investigate their utility for near-source emissions detection applications. Prototype SPods containing PID sensor elements from two different manufacturers yielded between 78% and 86% valid data over the study, producing a dataset of over 120,000 collocated pair fenceline measurements averaged into 5-min datapoints. Ten-second time-resolved SPod data from an elevated fenceline sensor signal day are presented, illustrating source emission detections from the direction of a facility 500 m west of the monitoring site. An SPod-triggered CGS acquired in the emission plume on this day contained elevated concentrations of 1,3-butadiene and cyclohexane (36 parts per billion by volume (ppbv) and 637 ppbv, respectively), compounds known to be emitted by this facility. Elevated concentrations of these compounds were observed in a subset of the 61 manual and triggered CGS grab samples acquired during the study, with winds from the west. Using novel wind-resolved visualization and normalization approaches described herein, the collocated pair SPod datasets exhibited similarity in emission source signature. With winds from the west, approximately 50% of SPod readings were above our defined theoretical detection limit indicating persistent measurable VOC signal at this site. Overall, this 19-month study demonstrated reasonable prototype SPod operational performance indicating that improved commercial forms of lower cost PID sensors could be useful for select VOC fenceline monitoring applications.

## 1. Introduction

In the United States (U.S.), there is growing interest in facility fenceline monitoring applications that improve understanding of industrial air pollutant emissions and help protect public health [1,2,3,4,5]. Technical advancements in lower cost sensors, higher performance field instruments, and data analysis are enabling new fenceline monitoring approaches [6,7,8,9,10,11,12,13,14,15]. Fenceline sensors typically provide real-time measurements of wind direction and pollutant concentrations to detect and identify abnormal source emissions. Most fenceline sensors have low site infrastructure requirements, (e.g., can be solar powered), but are limited in measurement sensitivity and compound speciation capability [13,16,17]. Conversely, emerging fenceline instruments possess higher sensitivity and accuracy as well as the ability to speciate specific toxic compounds in air, but require significantly more capital and operational investment [8,18,19,20,21,22,23,24,25,26].

One type of low cost fenceline sensor system is based on passively ventilated 10.6 eV PID sensor elements that can detect a subset of VOCs in advected emission plumes. While 10.6 eV PIDs encompass a range of air pollutant response factors [27], they are particularly sensitive to certain hazardous air pollutants (HAPs) [28], such as benzene and 1,3-butadiene. For the most sensitive 10.6 eV PID sensor elements, manufacturers advertise 0.5 ppbv detection capability for the reference gas isobutylene (defined PID response factor of 1.0), with an upper range typically of 2000 to 5000 ppbv, depending on model and configuration [29,30]. In practice, the field detection limit, accuracy, and unit to unit precision of the monitoring system will depend on the application and overall package design. The lower cost fenceline sensor prototypes described here represent the least-controlled instrument scenario where the passively ventilated PID sensor elements are directly exposed to ambient air and experience continuously changing temperature and relative humidity conditions that can affect sensor performance [31]. For exposed sensor elements, physical interferences such as condensation, dust, and insects can create significant precision, accuracy, and baseline drift issues. Partial engineering solutions such as heated PID sensor elements and custom data processing methods (algorithms) that remove baseline drift can improve fenceline sensor monitoring performance, with the latter approach described here.

As part of a larger measurement technology development project [13], prototype versions of the U.S. Environmental Protection Agency (EPA) SPod fenceline sensor and CGS system were deployed at a site located near chemical facilities in west Louisville, KY for a 19-month period. In this paper, we describe the results of the long-term study, which utilized two collocated SPods fitted with 10.6 eV PID sensors elements from two different manufacturers. Fenceline sensor data analysis approaches are detailed and an illustrative example of 10 s time-resolved emission plume detections with an SPod-triggered CGS is provided. Focusing on the compounds 1,3-butadiene and cyclohexane, known to be emitted from a nearby facility, we discuss 61 acquired CGS grab samples using SPod-measured wind data to inform the results. We analyze the overall long-term dataset consisting of over 120,000 5-min average collocated pair SPod measurements using novel wind-resolved visualization and normalization approaches. We describe the operational robustness and emission detection performance of the prototype SPods with a view towards the viability of improved commercial VOC sensors for fenceline applications.

## 2. Materials and Methods

### 2.1. Measurement Site

**Figure 1:**
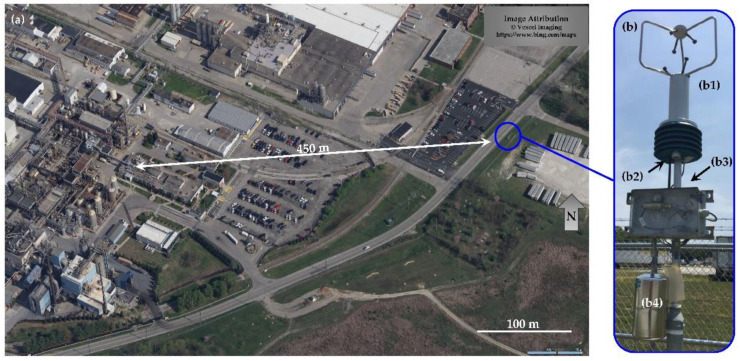
(**a**) Partial view of chemical facilities and sensor site (blue circle) where two SPods were located with white arrow indicating approximate direction of facility producing subsequently described SPod signal; (**b**) EPA SPod system with (**b1**) 3-D sonic anemometer, (**b2**) PID sensor, (**b3**) CGS trigger system, (**b4**) 1.4-L CGS.

Measurements were collected from 4 June 2018, to 5 January 2019, at a secured site in an open area within 1 km of multiple industrial facilities and terminal operations in west Louisville, KY (38.209694 N (latitude) and −85.842542 W (longitude), Figure 1a). This site primarily serves as a testing platform for EPA prototype and early commercial VOC sensors and does not contain regulatory-grade measurements. The nearest advanced monitoring system is located at the Louisville Air Pollution Control District’s (LMAPCD’s) Algonquin Parkway Air Monitoring Site (38.233661 N (latitude) and −85.766850 W (longitude)), with further details on collaborative measurements and overall project design contained elsewhere [13].

### 2.2. EPA SPod Sensors

Two prototype EPA SPod sensor packages were collocated at the measurement site. The SPods were fitted with three-dimensional (3-D) sonic anemometers (81000V, R.M. Young, Inc., Traverse City, MI, USA) producing high-resolution wind measurements (Figure 1(b1)). Each SPod in the collocated pair used high sensitivity (0.5 ppbv class) 10.6 eV PID sensor elements, either a MiniPID2-HS, red electrode (Ion Science Inc., Stafford, TX, USA) [29] or a Baseline^®^ piD-TECH^®^ eVx™, 045-014 (Ametek Mocon Inc., Minneapolis, MN, USA) [30], here after referred to as SPod1 and SPod2, respectively (Figure 1(b2)). The compound-specific response factors for this class of 10.6 eV PID sensors indicate detection sensitivity relative to isobutylene [27]. The SPods recorded time-synchronized VOC signal and wind data at a 1 Hz measurement rate. Each SPod contained an onboard Arduino^®^-compatible Teensy 3.2 microcontroller (Adafruit Industries, New York, NY, USA) that digitized the PID output voltage at 16-bit resolution over a range from approximately 0 to 5000 ppbv (isobutylene). Further information on the SPod design and communication features is described elsewhere [13]. Depending on the PID sensor type and condition, a 1000 ppbv isobutylene reference gas produced from 6000 to 10,000 digitized signal counts (cts), with SPod1 typically exhibiting about 20% higher relative response as compared with SPod2. Although the utilized PID sensor elements are capable of 0.5 ppbv detection sensitivity, the prototype SPod fenceline sensor packages typically exhibited minimum detectable concentrations in a range from 5 ppbv to 40 ppbv (isobutylene). With no VOC source emission plume present, typical SPod baseline signals levels were in the 1000 cts to 3000 cts range and reflected the convolution of slowly varying VOC air shed signal, electronic offsets, and variable artifact PID signal caused by temperature and relative humidity effects (baseline drift). Due to variable baseline effects and unknown composite PID response factors for detected emission plumes, the SPod PID signals are expressed as signal cts for this study and were not converted to ppbv. To help ensure PID functionality, the sensors were periodically checked in the field with short duration (~15 s) 500 ppbv isobutylene bump tests typically producing from 2000 to 5000 cts over baseline, depending on sensor type and conditions.

To reduce baseline drift caused by humidity and temperature changes, the PIDs were wrapped with strip heaters (PN HK6903, Minco Minneapolis, MN, USA), continuously operating at 10–15 °C above ambient temperature. This was an advancement over the early SPod prototypes which exhibit significantly more baseline drift [16]. Using an SPod or manually triggered canister acquisition system (Figure 1(b3)), 30 to 40-s duration 1.4 L CGS grab samples (PN 29-MC1400SQT, Entech Instruments, Simi Valley, CA, USA) (Figure 1(b4)) were collected and analyzed for over 100 individual chemical species by the EPA VOC Laboratory using method EPA TO-15 [32]. A total of 61 QA-valid CGS grab samples were acquired at this site with 34 of these manually collected under randomly encountered atmospheric conditions during site maintenance visits, without consideration of instantaneous SPod PID signal levels. There were 24 CGSs acquired by automatic SPod-trigger and three 1-min duration canisters were triggered by an experimental prototype field gas chromatograph that was collocated for part of the study [13]. User-set SPod trigger thresholds for CGS collections ranged from 4000 cts to 7000 cts depending on sensor type and system condition.

A total of five unique SPods were used to form the collocated SPod pairs over the course of the study. Two SPods contained Ion Science PIDs and three SPods used Ametek Mocon PIDs (Table S1). The first SPod1 unit deployed (SPod1a) had a relatively short lifespan of 120 days before PID failure and there was a 63-day delay in replacement of this unit contributing to the difference data completeness. The last SPod2 unit (SPod2c) had a nonoperational anemometer and wind data from the collocated SPod1b were used for both units during this 34-day period. The sharing of anemometer data for this period artificially enhanced the number of QA-valid days for SPod2 by about 6.5%. This dataset adjustment improved PID comparison coverage and signal origin information with little other impact, due to the high degree of similarity in collocated 3-D sonic anemometer data.

### 2.3. Data Analysis

SPod PID and wind data were analyzed using a program developed in R [33,34]. Automated quality assurance (QA) screening of PID values were first performed to identify periods of sensor malfunction (e.g., off-scale high values, unrealistically repeating values, or missing data) or measurements collected during rapid relative humidity changes that could produce signal artifacts. Native 1 Hz data that passed the automated QA-screen were aggregated to 10-s mean values and the baseline correction algorithm was applied to separate baseline drift and background VOC levels from rapid signal changes indicative of advected plumes from local emission sources. The baseline correction algorithm employed in this study fit a smoothed quantile regression to the slow drift signal pattern and subtracted away area under this curve to remove the non-plume signal [35] (Figure S1). The final QA assessment of the QA-valid dataset included the removal of data with artifactual 3-D sonic anemometer results (e.g., spikes larger than 10 m/s, or repeated values) and visual inspection of data trends for PID sensor functionality.

As described in Results, the coupled 10-s baseline-corrected SPod PID and wind data allowed elevated VOC events to be investigated with high time resolution. Additionally, these data assisted in sensor performance assessment on a daily basis by allowing VOC source-impacted 10-s periods to be identified and excluded from automated sensor noise calculations in a multi-step screening process. As a first step, the median value of the collection of all baseline-corrected 10-s periods (up to 8640 values) for each day was calculated. All 10-s periods with baseline-corrected PID values less than 4 times this median formed a sensor noise calculation subset. For each day, the daily sensor noise (σ*_n_*) was defined as the standard deviation of this subset. As a second step in the screening process, any 10-s baseline-corrected SPod PID value exceeding 5 times (σ*_n_*) was defined as potentially source-impacted and was excluded from subsequent aggregated 5-min sensor noise calculations.

For primary data summary, all QA-valid baseline-corrected 10-s data were aggregated to sequential 5-min time periods (N = 288 max for one day), with means and standard deviation (σ*_i_*) of each 5-min period calculated. A theoretical detection limit (TDL) was based on the collection of QA-valid 5-min periods that did not contain source-impacted signal [i.e., no 10 s periods > 5 times (σ*_n_*)]. A daily median of the collection of non-source-impacted σ*_i_* was calculated and the TDL was defined as three times this value. This TDL typically presents an optimistic view of emission plume detection capability, as it considers only a low estimate of the sensor noise floor and does account for baseline drift, emission plume to sensor coupling efficiency, low end detector responsivity, or sampling interferences. Since the TDL definition excludes source-impacted time periods, it relies on the presence of a statically representative set of low σ*_i_* time periods each day. All QA-valid 5-min periods with mean PID values exceeding the TDL were labeled as “in detection” or data “above TDL” while all other periods below were labeled as data “below TDL”. Alternate definitions of TDL are discussed in results and Supporting Information.

## 3. Results and Discussion

### 3.1. Example of Time-Resolved Data from an Elevated Source Signal Day

**Figure 2:**
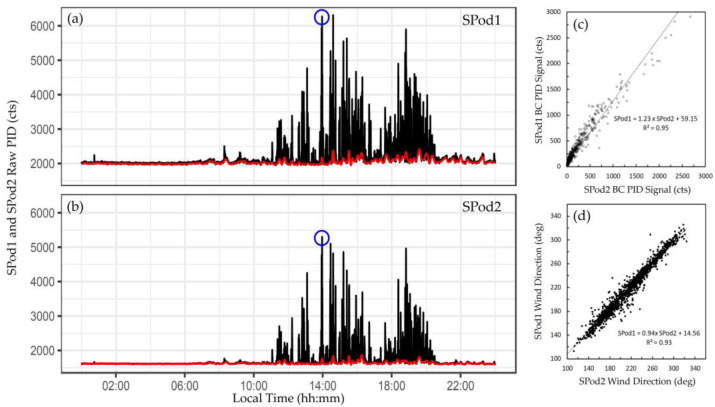
Example of collocated SPod data from 7/17/19: (**a**,**b**) SPod1 (top panel) and SPod2 (bottom panel), 10 s average timeseries (black traces) with superimposed baseline correction fits (red traces), blue circles indicate expanded view during CGS acquisition shown in Figure 3, (**c**) SPod PID comparison of 1-min background-corrected data, (**d**) SPod wind direction comparison of 1-min data.

An example of 10-s time resolved SPod data from an elevated source signal day is provided in Figure 2a,b. The calculated baseline correction (red line) was subtracted during data processing. In this example, the baseline correction procedure had a minor effect on the raw data as the signal levels were relatively high as compared with the baseline drift (an alternative example is provided in Figure S1). Correlation of wind data and PID signal between the collocated SPods, similar to that shown in Figure 2c,d, was typically observed on high signal days where SPod PID levels were well above the TDL. With a slope of 1.23, the generally higher responsivity of SPod1 was observed in Figure 2c, with some nonlinearity in comparison at higher values noted. The agreement in wind data between the SPods was due in part to the high performance 3-D sonic anemometers utilized in the EPA SPod design (approximately 50% of system cost). These robust wind data findings may not translate to emerging commercial SPod-type sensors, which could employ lower performance (and lower cost) anemometers. The slope of the regression line of Figure 2d indicates a slight angular misalignment between the SPod anemometers. Inaccuracies in the absolute alignment of fenceline sensors produces additional uncertainty in back-trajectory models, especially at greater sensor to source separation distances.

**Figure 3:**
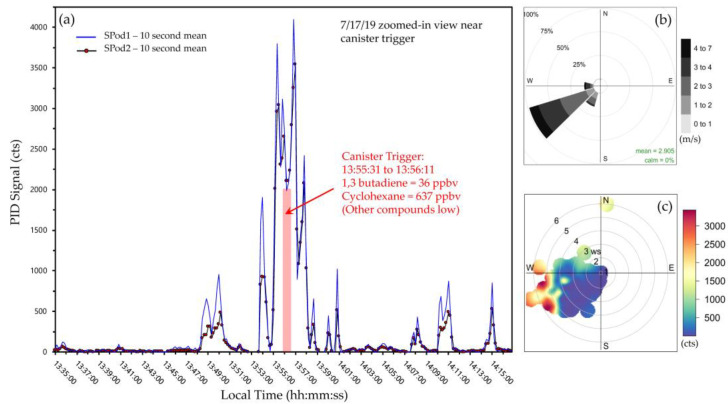
Example of collocated SPod data and canister trigger time frame on 7/17/19: (**a**) SPod1 (blue line) and SPod2 (red points trace) baseline-corrected 10 s average timeseries with canister trigger duration indicated by red rectangle and described in red text; (**b**) SPod1 and SPod2 combined wind rose; (**c**) SPod1 and SPod2 combined SDI plot showing the wind-resolved interpolated median concentration of PID signal.

The blue circles in Figure 2a,b indicate the occurrence of a CGS automated acquisition from one of the SPod units, with an expanded view of the sample time period shown in Figure 3. The primary period of elevated signal in this expanded time window was approximately two minutes in duration and was detected by both SPods (Figure 3a). The modulated nature of this signal was typical and was due to wind direction changes that determine the spatial overlap of the sensors and the advected source plume. The source emission is believed to be relatively constant in this case due to the temporally sustained signal envelope from ~11:00 to 21:30 local time (Figure 2a,b). The wind rose (Figure 3b) and SPod source direction indicator (SDI) plot (Figure 3c), indicate that the origin of the observed PID signal was from the direction of a facility to the west, similar to the direction of the white arrow shown in Figure 1. The wind-resolved signal of Figure 3 (13:35 to 14:15 local time) resemble the daily trend (Figure S2). The laboratory-determined concentration of 1,3-butadiene and cyclohexane for the SPod triggered 40-s CGS grab sample were 36 ppbv and 637 ppbv, respectively, with other compounds present at much lower levels typical of this airshed. Since the facility to the west was known to emit 1,3-butadiene and cyclohexane, the combination of the SDI plot and the CGS data support general conclusions on source origin for the time period of Figure 3. However, the limited time CGS data do not inform the speciation of the broader signal envelope (Figure 2a,b and Figure S2) observed on this day. 1,3-butadiene and cyclohexane are both easily detected by 10.6 eV PID with sensor response factors of 0.8 and 1.3, respectively [27].

### 3.2. CGS Grab Samples and Coincident SPod Data

**Figure 4:**
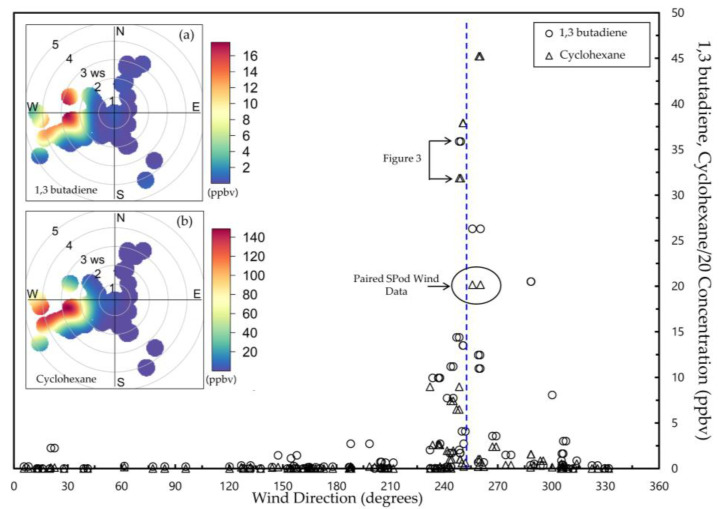
Concentrations of 1,3-butadiene (circles) and cyclohexane divided by a factor of 20 (triangles) from 61 CGS samples plotted by SPod-measured wind direction (typically two wind data points per CGS value). CGS data of Figure 3 are indicated. Modified SDI plots [inset (**a**,**b**)] combine wind information from both SPods and replace PID cts values with CGS ppb data for (**a**) 1,3-butadiene and (**b**) cyclohexane, utilizing a median statistic for interpolation. Blue dashed line corresponds to direction indicated by white arrow in Figure 1. The uncertainty of the TO-15 concentration measurements is ±30%.

A total of 61 QA-valid CGS grab samples were acquired at this site and analyzed by EPA method TO-15. Although the TO-15 analysis produced concentration measurements of approximately 120 VOCs, we focus here on the compounds 1,3-butadiene and cyclohexane as they are particularly important HAPs in the airshed based on facility emissions profiles [13]. Measured concentrations for 1,3-butadiene and cyclohexane, were plotted by wind direction for both SPods, typically generating two slightly offset wind data points per CGS concentration value (Figure 4). Here, the cyclohexane concentration is divided by a factor of 20 for ease of viewing. The inset in Figure 4 show modified SDI plots that use the SPod wind directions (SPod1 and SPod2 combined), but the PID readings are replaced with laboratory-determined 1,3-butadiene (Figure 4a) and cyclohexane (Figure 4b) CGS concentrations. The results showed elevated concentrations of 1,3-butadiene and cyclohexane at this site with wind directions between 240 and 270 degrees for multiple CGS samples acquired during the study. This general wind direction range implied source emissions from the facility to the west (white arrow of Figure 1), which is known to emit these compounds. An alternative view of CGS data for these compounds by date of acquisition is given in Figure S3. The compounds 1,3-butadiene and cyclohexane dominated the speciated profiles in these elevated CGS cases. Other compounds, such as mixtures indicative of gasoline storage emissions, were elevated in some CGS samples and were associated with other source origin directions and atmospheric transport conditions.

### 3.3. Overview of the SPod Dataset

For primary data analysis, the 10-s time resolved data were aggregated to 5-min time periods. As summarized in Table 1, there were 167,328 available 5-min measurement periods over the 581 deployment days from 4 June 2018 to 5 January 2020. SPod1 units produced QA-valid data for 78.4% of the available periods data on 476 study days. SPod2 units produced QA-valid data for 85.6% of available periods over 521 study days. As described in Methods, the SPod2 data completeness would be 6.5% lower if replacement sonic anemometer data from SPod1 were not used. The overall percentage of QA-valid data greater than the TDL was 21.2% and 25.6% for SPod1 and SPod2, respectively. There was no apparent temporal trend in the TDL (Figure S4) or the detection rate (Signal Above TDL, Figure S5) for the longest running sensors.

Subsequent analysis focuses on time periods where collocated SPod pairs were simultaneously producing QA-valid 5-min data. This paired SPod dataset represents 120,656 data points from each SPod or 72.1% of the available the 5-min periods for the study. As compared with the overall QA-valid dataset, the paired dataset exhibited a similar percentage of above TDL at 20.7% for SPod1 and 25.3% for SPod2. The average [median] daily TDLs for the paired data were 53.2 cts [46.7 cts] and 12.8 cts [9.3 cts] for SPod1 and SPod2, respectively, with differences primarily due to sensitivity and noise variances between the PID sensor types. Figure S5 presents the range of daily TDL values. Besides the one SPod2 unit representing 50 QA-valid days, little difference in TDL between the deployed units within each sensor type was observed.

### 3.4. Source Directional Analysis of Paired SPod Dataset

**Figure 5:**
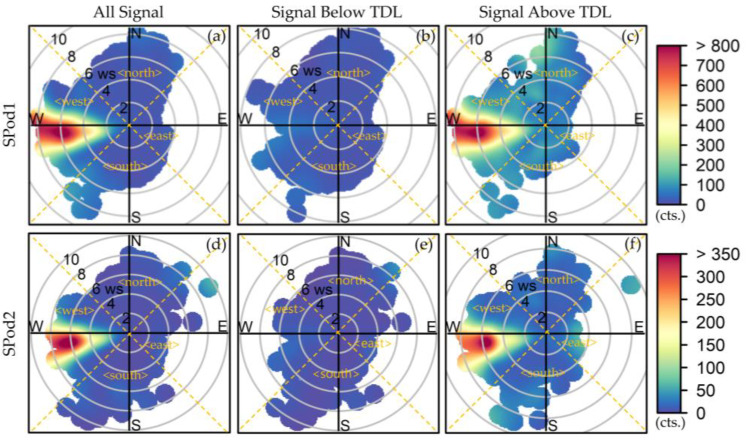
SDI plots for (**a**–**c**) SPod1; (**d**–**f**) SPod2, with (**a**,**c**) including all data consisting of 120,656 QA-valid paired 5-min periods, (**b**,**e**) a subset of data below the TDL, (**c**,**f**) a subset of data above the TDL. Forty-five-degree rotated quadrant indicated by dashed line and <direction>.

As shown in Figure 5a,d, the 120,656-value paired SPod dataset exhibited source signal origin direction similar to the previously described shorter-term examples. Here, the SDI plots are subset into above TDL and below TDL values to facilitate inter-sensor comparison and elucidate the TDL demarcation. Figure 5b,e represent 95,731 and 90,073 5-min values below TDL, while Figure 5c,f show 24,925 and 30,583 5-min values above TDL for SPod1 and SPod2, respectively. The below TDL values exhibit slight signal residuals reminiscent of the primary signal lobes in the above TDL case, providing support for a reasonable TDL definition, further discussed subsequently.

A 45-degree rotated quadrant system was defined (dashed lines of Figure 5a,d). In this representation, the <west> quadrant captures the primary signal lobe and the occurrences of wind direction and detections by quadrant are compared in Table 2. The fraction of SPod data in Figure 5 is summarized in Table 2 in each quadrant for all wind speeds (left panel) and for wind speeds greater than 1.0 m/s (right panel). For all wind speeds, although the <south> quadrant accounts for 40.1% and 36.8% of the data from SPod1 and SPod2, respectively, the <west> quadrant disproportionately represents signal above TDL, with about 50% of all detections coming from this direction. With the current TDL definition, between 49% and 56% of <west> quadrant data are above TDL (in detection), for SPod1 and SPod2, respectively.

For the subset of data with wind speeds above 1 m/s (Table 2, right panel), the percentage of detected signal from the <south> decreases markedly. This is due in part to plume transport effects and wind speed distributions. Additionally, the low wind speed portion of the PID signal was affected by the baseline correction approach utilized in this analysis that discounts slowly varying airshed signal in favor of directly advected source emission plume signal. With winds > 1 m/s, if only above TDL data are considered, data originating from the <west> comprised 81.7% and 73.8% of all detections from SPod1 and SPod2, respectively. As subsequently discussed, wind speeds from the <west> were generally elevated as compared with other quadrants, which is typical of prevailing winds in this area. It is noted that the <west> quadrant faces a two-lane roadway (Figure 1) with modest daily traffic volumes and vehicle wake effects could be a factor in wind speed and wind direction distributions. The PID detection signal is not believed to be significantly impacted from vehicle emissions, as there was no discernable diurnal pattern and traffic volumes on the roadway were low (Figure S7).

### 3.5. Analysis of Detection-Normalized SPod Dataset

**Figure 6:**
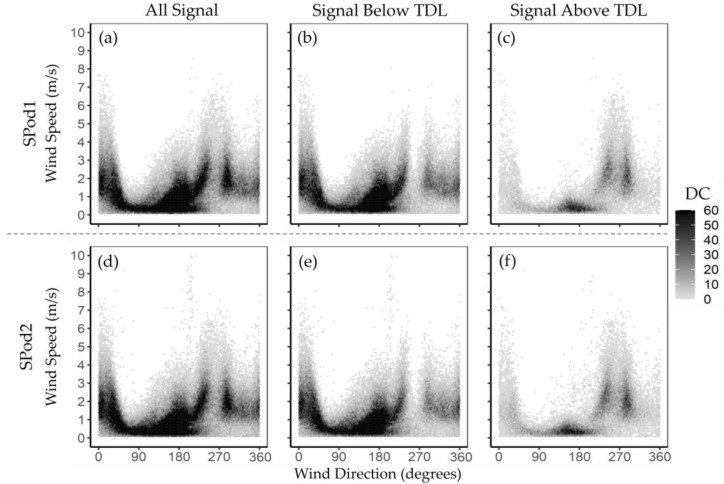
DC plots for (**a**–**c**) SPod1; (**d**–**f**) SPod2; with (**a**,**c**) including all data consisting of 120,656 QA-valid paired 5-min periods, (**b**,**e**), a subset of data below TDL; (**c**,**f**) a subset of data above TDL.

To further explore the observed SPod signal as a function of wind data, the paired dataset was transformed into what we call detection count (DC) matrices (Figure 6). The wind speed and wind direction of each dataset entry were separated into 0.1 m/s bins (0 m/s to 10 m/s) and one-degree bins (0° to 360°), respectively. Each dataset entry was assigned a unity value (1.0) and retained its below TDL or above TDL label. One purpose of the DC matrix is to examine source detection characteristics without bias effects associated with extreme PID readings, since each detection event is treated equally. Figure 6a,d show the DC matrices for all data, without regard to TDL, demarcation, for SPod1 and SPod2, respectively. Figure 6b,e shows DC plots for data below TDL and Figure 6c,f present data above TDL for each sensor. Similar results for the collocated SPod pairs were observed in all cases.

In the above TDL subsets, the presence of separate signal detection regions was elucidated benefitting from the binary form of the DC data. Source signal from the <west> quadrant at higher wind speeds was observed, as was potential signal around 180 degrees at lower wind speeds, tentatively attributed in part to tank farms approximately 700 to 800 m to the southwest of the site. As summarized in Table 2, detections from the <south> are present at low wind speeds and are ascribed in part to source emissions accumulating during calms, with these findings supported by the canister speciation data beyond the scope of this paper. The primary signal from the process unit to the west was disproportionally observed at wind speeds greater than 1 m/s, implying a partially elevated source emission may have contributed to this signal. The potential presence of multiple lobes in the signal from the west, one centered near 260 degrees and one near 280 degrees, could be a result of multiple sources, wind flow obstructions, or wake effects. This detail, as well as the location of the source, would be immediately informed by the addition of one or more spatially separated sensor locations for triangulation. Regarding potential effects of TDL definition (i.e., as 3 × σ*_i_*) on the above and below TDL demarcation and wind-resolved detection signal form, Figure S7 provides an alternative view of information in Table 2 and Figure 6 with higher TDL multipliers, although the percentage of detections in the DC distributions are similar, indicating a relatively robust result with little substantive dependency on definitional choices.

**Figure 7:**
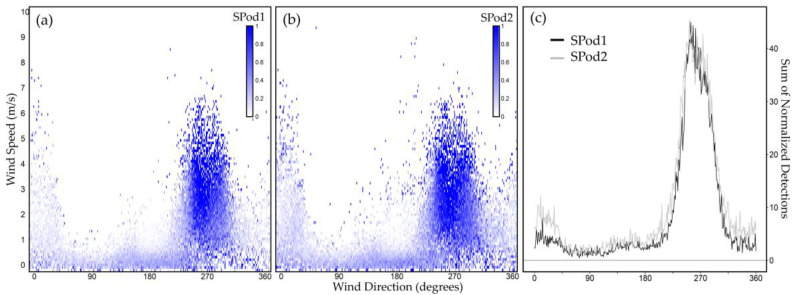
Wind direction and wind speed-resolved frequency of detection plots for (**a**) SPod1; (**b**) SPod2; (**c**) showing the cumulative detection probability for both SPods by wind direction.

The DC matrices may be further compared after a normalization step. In one approach, the above TDL subsets (Figure 6c,f) may be normalized by dividing the number of detections for each cell by the total number of data points acquired for that cell from the matrices containing All Data (Figure 6a,d). This normalization approach provides wind speed and wind direction-resolved detection frequency on a cell-by-cell basis (Figure 7a,b). The elevated source signal from the <west> quadrant is expected as many cells are near 100% detection probability in the primary source signal lobes. The reduction in detection probability moving away from 270 degrees is also expected as the observation moves away from the primary direction of source advection and the emission plume to sensor spatial overlap decreases. Due to overall data density, this normalized form visually discounts the low wind speed signal from the <south> described in Figure 6 and Table 2. To facilitate comparison across the collocated SPod pairs, these data were aggregated in wind speed by summing detection probabilities by individual wind degree bins to produce an angle-resolved source signal (Figure 7c). From this perspective, SPod1 and SPod2 exhibited good agreement regarding the primary source signal between 240 and 280 degrees.

Whereas the normalization approach of Figure 7 provides an absolute measure of angle-resolved detection density for a selected time range (e.g., daily to annual summary), the clarity of result (e.g., high degree of correlation in Figure 7c) is a consequence of elevated detection rates from the <west> quadrant (Table 2). These high detection rates were in part due to our TDL definition, but as described in Figure S7, the wind-resolved signal forms appear relatively insensitive to TDL multiplier for this dataset. In general, these consistent detections over time are atypical of shorter time-scale stochastic source emission fenceline measurements.

**Figure 8:**
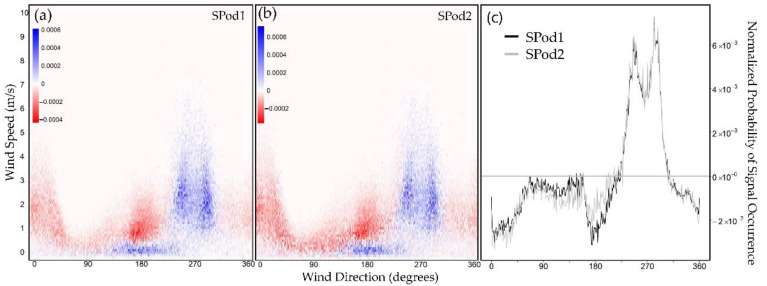
Individually normalized above TDL DC matrix minus all signal DC matrix for: (**a**) SPod1; (**b**) SPod2; (**c**) wind speed aggregated version of the angle resolved relative detection signal for both SPods.

Alternative DC matrix normalization approaches can be used to enhance source signal features for lower and/or variable source emissions or higher atmospheric dispersion conditions, where the total amount of data above TDL decreases. In another normalization approach, the all signal DC matrix (Figure 6a,d) and the above TDL subsets (Figure 6c,f) were individually normalized by their respective DC sums to produce separate probability of occurrence matrices for all observed data and data above detection limit. The subtraction of the self-normalized matrix containing all data (Figure 6a,d) from the matrix containing above TDL data (Figure 6c,f) produces a differential view of wind-resolved relative detection signal (Figure 8a,b). As the proportion of data above TDL decreases as part of the whole, this normalization form exaggerates the source signal pattern to an increasing degree, so this interpretation must be used in conjunction with the more standard normalization of Figure 7 as well as other QA and signal comparison metrics. In contrast to Figure 7, the low wind speed signal from the <south> is visually enhanced, as is the bifurcation in the primary signal lobe to the <west>. This form was collapsed into wind speed (Figure 8c), to investigate the correlation between deployed sensors over long time scales with a natural source signal threshold near zero differential probability. This normalization form is independent of both the total amount of observed data and the proportion of data above TDL. Therefore, it can be applied on a variety of timescales and conditions to search for similarity in source signatures. This normalization form can provide complementary metrics to standard normalization to be used in future envisioned source detection approaches based on signal pattern recognition. These normalized DC detection space calculations augment similar analyses using fully calibrated sensor data which carries source strength information.

## 4. Conclusions

In this paper, we describe a 19-month demonstration of prototype 10.6 eV PID fenceline sensors and CGS systems deployed at a site near chemical facilities in Louisville, KY. The purpose of this project was to evaluate the performance of this class of lower cost near-source monitoring approaches and improve understanding of their potential to support emerging fenceline monitoring applications. This analysis considered the detection capability of the sensors for directly advected plumes and utilized a baseline correction approach that removed slowly varying sensor drift and some portion of VOC air shed signal. This analysis was limited to fenceline applications (500 m from the source) and is not intended to inform the use of these technologies in community deployments where sensitive, calibrated, and stable VOC measurements are required as these locations are further from the emission source.

This project demonstrated that collocated SPod sensors using PIDs from different manufacturers can produce useable data that informs proximate emission sources. This study produced over 120,000 QA-valid paired 5-min data points from the collocated SPod pairs. These pairs agreed in the assessment of wind-resolved source signal and were supported by acquired CGS data. Canister data acquired with winds from the direction of the facility the west exhibited instances of elevated 1,3-butadiene and cyclohexane compounds known to be emitted by this facility. The study showed consistent SPod PID signal above our defined TDL with winds from the west. However, the detected PID signal cannot be assumed to be solely composed of 1,3-butadiene and cyclohexane, and certainly includes other VOC compounds that may be emitted by the facility and potentially other sources. The study demonstrated angle-resolved signal analysis that was useful in separating fenceline senor signal characteristics to isolate suspected source emissions.

Details of the QA procedures, TDL calculations, and novel sensor signal visualizations were described. Whereas this prototype version of the SPod fenceline sensor exhibited about 20% data loss due to component failures and delays in replacements, envisioned commercially available versions of the technology should be more robust, weather-tight, and exhibit improved data completeness. Future work in this area will continue to improve QA and data analysis approaches while implementing calibrated PID measures and exploring multiple point source triangulation as well as inverse emission estimate approaches.

## Figures and Tables

**Table 1:** Summary of the 5-min SPod Dataset.

Data Summary	SPod1	SPod2
All Available 5-min Periods in Time Frame [Days]	167,328 [581]	
Final QA-valid 5-min Periods [Study Days Represented]	131,120 [476]	143,242 [521]
Percentage of QA-valid 5-min Periods [Percentage of Study Days Represented] (%)	78.4 [81.9]	85.6 [89.7]
Percentage of QA-valid Data > TDL (%)	21.2	25.6
Paired 5-min Periods [Study Days Represented]	120,656 [445]	
Percentage of Paired 5-min Periods [Percentage of Study Days Represented] (%)	72.1 [76.6]	
Percentage of Paired Data > TDL (%)	20.7	25.3
Average [Median] of Paired Data Daily TDL (cts)	53.2 [46.7]	12.8 [9.3]
Minimum [Maximum] of Paired Data Daily TDL (cts)	22.8 [425.1]	4.1 [75.9]

**Table 2:** SPod QA-valid and paired data, 45-degree rotated quadrant analysis, (top panel) All data; (bottom panel) subset of data above 1 m/s wind speed.

	All Wind Speeds			
Quadrant (SPod1, SPod2)	Total 5-min Periods	Percentage of Total (%)	Percentage of Total > TDL (%) ^1^	Percentage in Each Quadrant > TDL (%) ^2^
<west>	27,216	22.6	53.0	48.5
<north>	28,344	23.5	9.2	8.0
<east>	16,655	13.8	8.2	12.3
<south>	48,441	40.1	29.7	15.3
<west>	26,352	21.8	48.5	56.3
<north>	30,140	25.0	14.0	14.2
<east>	19,782	16.4	10.6	16.4
<south>	44,382	36.8	26.8	18.5
	**Wind Speeds > 1.0 m/s**			
**Quadrant** **(SPod1, SPod2)**	**Total 5-min Periods**	**Percentage of Total (%)**	**Percentage of Total > TDL (%)** ^1^	**Percentage in Each Quadrant > TDL (%)** ^**2**^
<west>	23,267	33.1	81.7	50.7
<north>	22,575	32.2	9.8	6.3
<east>	3526	5.0	0.7	2.9
<south>	20,844	29.7	7.8	5.4
<west>	22,337	32.6	73.8	59.1
<north>	23,612	34.4	16.6	12.6
<east>	3703	5.4	1.0	4.6
<south>	18,912	27.6	8.6	8.1

^1^ “Percentage of Total > TDL” refers to count of data above TDL in that quadrant divided by the count of data above TDL from all quadrants. ^2^ “Percentage in Each Quadrant > TDL” refers to count of data above TDL in that quadrant divided by total count in that quadrant.

## Data Availability

Dataset and data dictionary are provided at https://catalog.data.gov/dataset/epa-sciencehub (accessed on 14 January 2022) under EPA SPod Fenceline Monitoring Rubbertown Dataset.

## Supplementary Materials

The following supporting information can be downloaded at: https://www.mdpi.com/article/10.3390/s22093480/s1, Table S1: SPod sensor descriptions; Figure S1: Baseline correction procedure; Figure S2: Daily wind pattern on 17 July 2019; Figure S3: Grab sampling results by date; Figure S4: Distribution of TDLs; Figure S5: Long-running sensors and detection levels Overtime; Figure S6: Daily distribution of PID values above TDL; Figure S7: Other possible TDL threshold levels.
