# Supplementary material for: Demonstration of VOC Fenceline Sensors and Canister Grab Sampling near Chemical Facilities in Louisville, Kentucky
Source: Sensors (Basel). 2022 May 3;22(9):3480. doi: 10.3390/s22093480 (PMC9103096; doi:10.3390/s22093480)
Supplement: Supplementary file 1 [file sensors-22-03480-s001.zip › sensors-1685739-supplementary.pdf]

Supplementary Information for Demonstration of Fenceline VOC Sensors and Evacuated Canister Sampling near Chemical Facilities in Louisville, Kentucky

Megan MacDonald*, Eben Thoma†, Ingrid George, and Rachelle Duvall
U.S. Environmental Protection Agency, Office of Research and Development, Center for Environmental Measurement and Modeling, RTP, NC, USA
*Oak Ridge Institute of Science and Engineering Fellowship Program
†Corresponding Author

Supplementary Information Table of Contents

Table S1: SPod Sensor Descriptions 1
Figure S1: Baseline Correction Procedure 2
Figure S2: Daily Wind Pattern on 17 July 2019 3
Figure S3: Grab Sampling Results by Date 4
Figure S4: Distribution of TDLs 5
Figure S5: Long-Running Sensors and Detection Levels Overtime 6
Figure S6: Daily Distribution of PID values Above TDL 7
Figure S7: Other Possible TDL Threshold Values 8
References 8

Table S1: SPod Sensor Descriptions

Table S1: SPod sensor specifications and deployment descriptions from 19-month study.

SPod ID	PID type	Deployment Dates (mm/dd/yy)	Total 5-min Periods and [Days]	Percentage of QA-valid 5-min Periods (%) and [Days]	Percentage of QA-valid 5-min Periods >TDL (%) and [Days]	SPod Equipment Note and [Date]
SPod1a	Ion Science	6/4/18 – 10/1/18[1]	34,163 [120]	47.5 [58]	16.6 [58]	PID Fail [7/31/18][1]
SPod1b	Ion Science	10/2/18 – 1/5/20	121,132 [422]	94.8 [418]	21.8 [387]	Study End [1/5/20]
SPod2a	Baseline-Mocon	6/4/18 – 9/6/19	129,344 [451]	94.1 [439]	27.7 [363]	PID Fail [9/6/19]
SPod2b	Baseline-Mocon	10/5/19 – 11/28/19	14,719 [52]	86.8 [50]	8.2 [50]	PID Fail [11/26/19]
SPod2c	Baseline-Mocon	12/3/19 – 1/5/20	9,504 [33]	92.0 [32]	21.4 [32]	Anemometer failure[2]

[1]SPod1a PID failed on 7/31/18 and unit was not replaced for 63 days causing loss in QA paired data (SI Figure 1).
[2]The anemometer of SPod2c malfunctioned at deployment and wind data from SPod1b was used for this 34-day period.

Each collocated SPod pair consisted of one unit from SPod1, fitted with Ion Science PID, and one unit from SPod2, which used a Baseline Mocon PID. Periodic field failures of the prototype SPod components were observed as described in Table S1 and the units experienced downtime due to delays in field replacement. For example, the PID Sensor of SPod1a failed on 7/31/18 and it required 63 days before unit SPod1b could be installed in the field as a replacement. These delays in replacement were largely responsible for the differences in percentage of QA-valid periods between the sensor types. SPod2c

experienced a sonic anemometer failure at the beginning of its deployment. Due to the similarity in sonic anemometer data between the SPods throughout the study, we utilized the time-aligned wind data from SPod1b for this 34-day period to strengthen the PID-to-PID comparison. The "days" count of Table S1 indicates the number of unique days represented in the specified data subset. SPod2b exhibited a higher noise level compared to other units using the same PID sensor, which resulted in a much lower percentage of above TDL data points from this unit. While all operation days of SPod2a contained some values above the TDL, the performance of this unit was dissimilar to other units using the same sensor type. Figure S4 provides additional information on individual SPod noise levels.

Figure S1: Baseline Correction Procedure

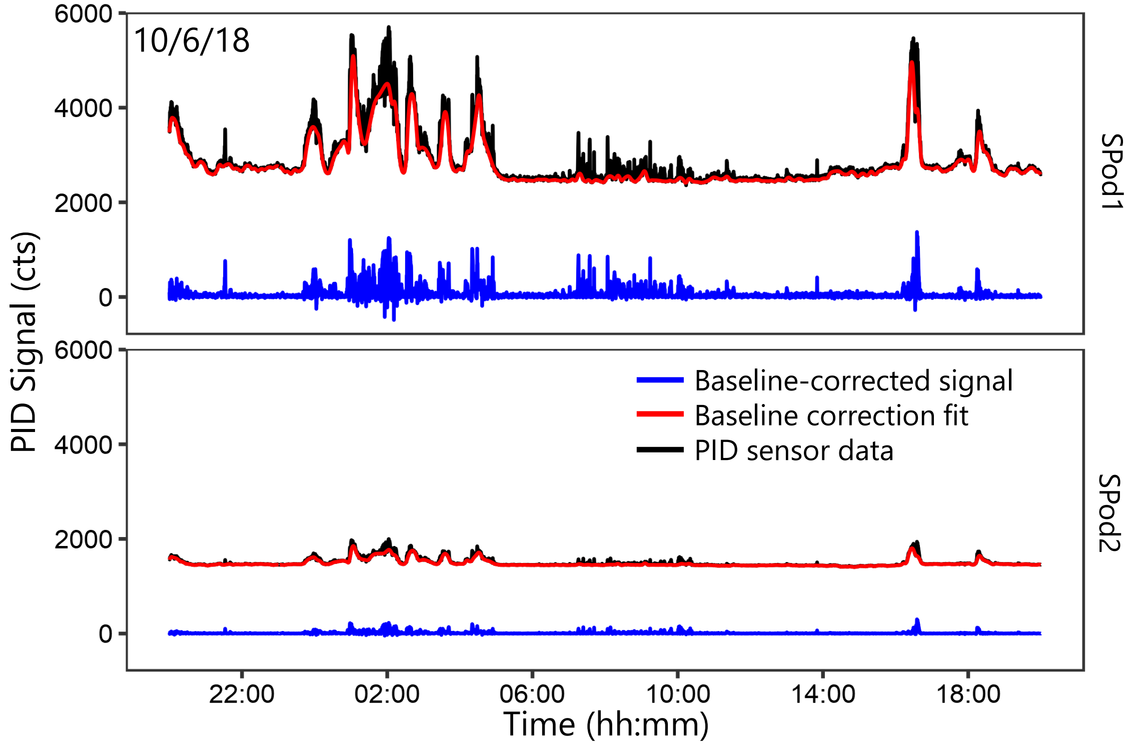

Figure S1: Signal data collected on 10/16/18 with temporal baseline correction (red trace) fit to PID raw sensor readings (black trace) then subtracted to produce baseline-corrected signal (blue trace) for SPod1 (top panel) and SPod2 (bottom panel).

The first-generation baseline correction algorithm (red trace of Figure S1) used in this analysis determines the slowly varying portion of the signal envelope from signal of interest using the "getbaseline" function in the detrendr R package [1]. This algorithm estimated and filtered trends by fitting a quantile regression to multiple windows in the signal trace. The temporal response of baseline correction spline fit was set so that it excludes the rapidly varying advected source emission plume signal that is driven on and off the sensor by meandering winds. The baseline fit was then subtracted from the raw signal to form a new near-zero baseline. This removes the slowly varying portion of the raw signal while preserving the rapidly varying advected plume signal. This type of time-based correction approach is useful for near-source or fenceline sensor data where detection of source plumes is the primary monitoring objective. In addition to

removing slowly varying sensor baseline drift (sensor bias), this approach also removes slowly varying background VOC airshed signal, so it is not appropriate for applications where absolute accuracy is required[2]. As opposed to the example provided in Figure 2 of the text, Figure S1 shows a larger amount of slowly varying signal overnight periods due in part to VOC accumulation under calm wind conditions. Other baseline correction algorithms are possible and in development, such as calculations based on relative humidity or temperature, and are likely more appropriate for non-fenceline applications associated with PID baseline elevations. The first-generation baseline correction approach utilized here produced slight negative overshoots after plume subtraction in some cases, which can be seen in the blue trace of Figure S1 (particularly in SPod1). These artifacts were believed to have little impact on study results.

Figure S2: Daily Wind Pattern on 17 July 2019

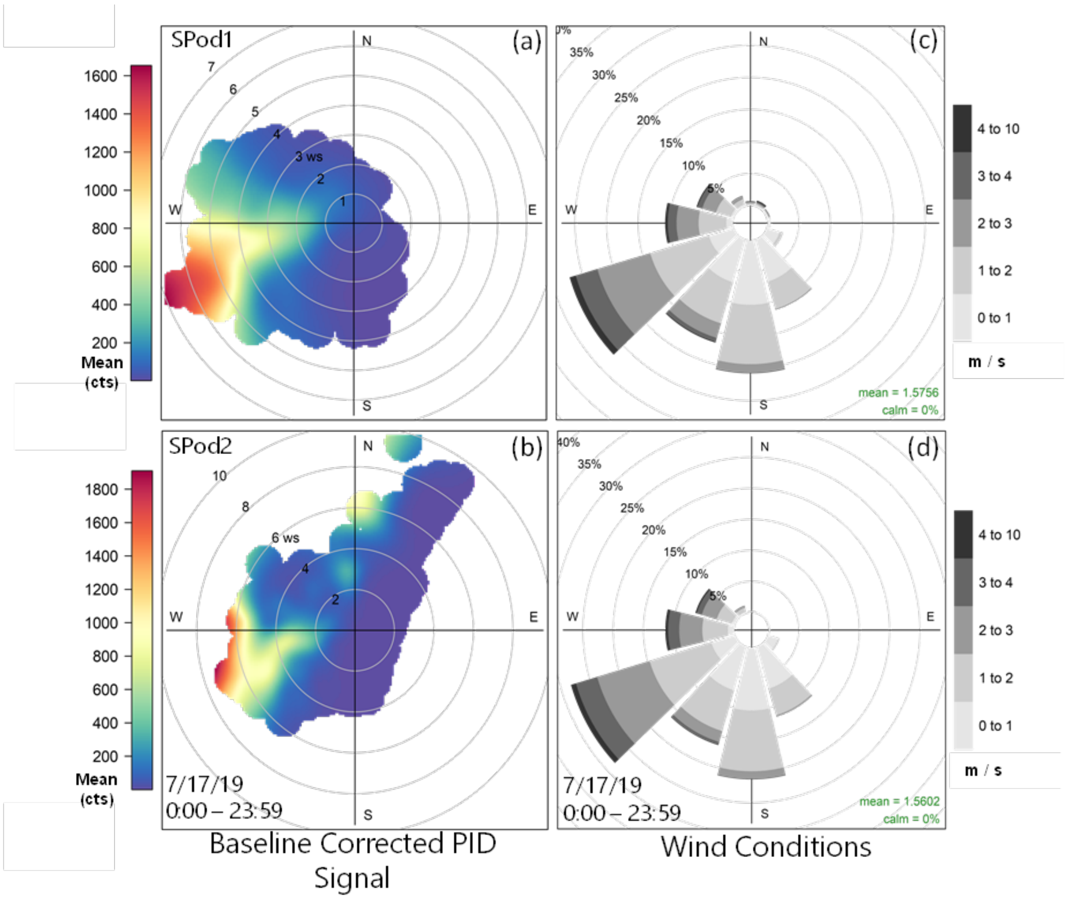

Figure S2: SDI plots for (a) SPod1 and (b) Spod2 and wind frequency roses for (c) Spod1 and (d) SPod2 (c-d) on 7/17/19.

Daily plots for 7/17/19 indicated wind primarily from the south and southwest. Figure 3 in the text is limited to the time frame of when a canister acquisition took place, between 13:35:00 and 14:15:00 on that day. Figure S2 shows the overall day contains more wind from the south than the narrow view in Figure 3. Unlike Figure 3, where the sensors are combined into one SDI plot and wind graph, Figure S2 shows these plots for each individual sensor in the collocated pair. Both sensors agree that the highest signal occurs when winds shift from the south to the west and carry air from the adjacent facility to the sensors'

location. The SDI plots shown in Figure SI2 and elsewhere in this analysis are bivariate (wind speed and wind direction) plots that utilize a smoothing function to present a continuous image of a pollutant and are built using the Openair R package [3].

Figure S3: Grab Sampling Results by Date

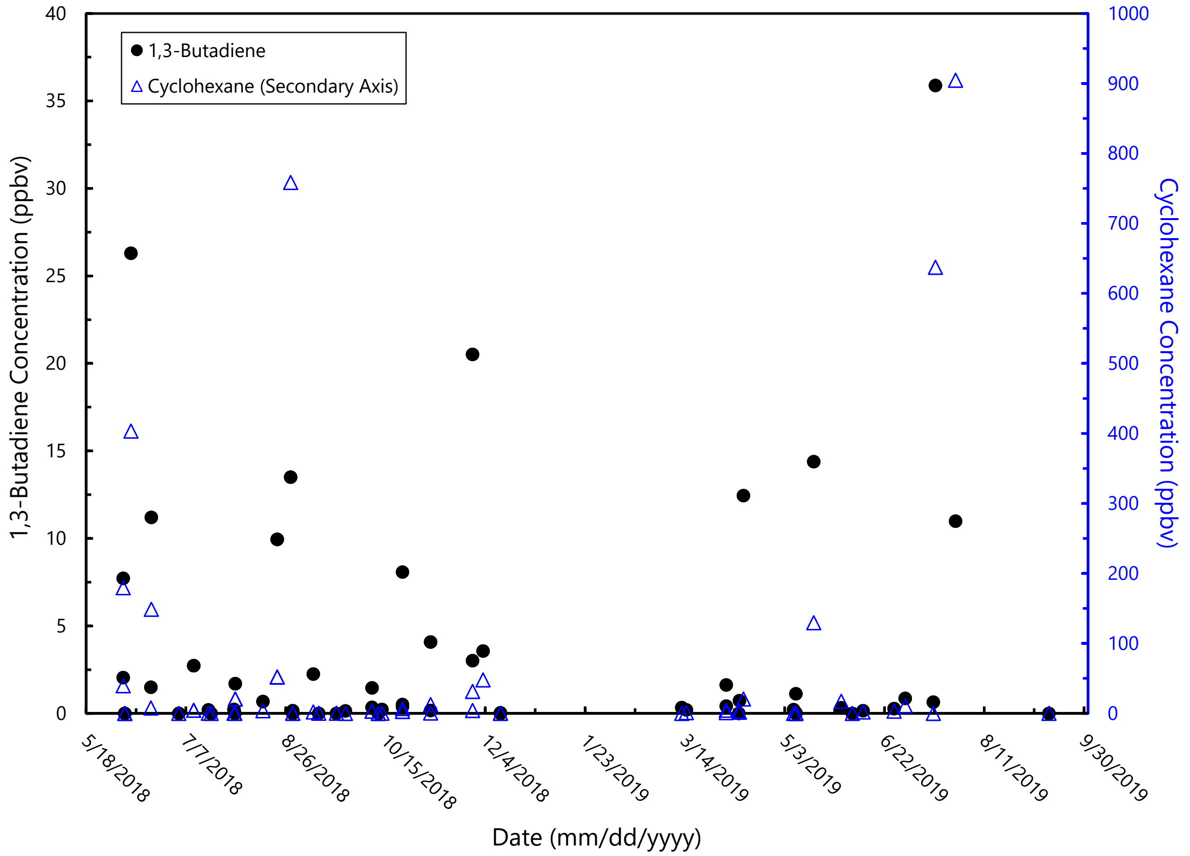

Figure S3: Measured compounds of 1,3-butadiene (black circles) and cyclohexane (blue triangles, secondary axis) from EC grab canister sampling completed throughout duration of study.

These elevated samples associated with winds from the west are not from specific times of the year but scattered throughout the study as shown in Figure S3. This graph presents the 61 QA-valid EC samples of Figure 4 in the text by date of acquisition to illustrate the temporal distribution of elevated 1,3 butadiene and cyclohexane samples observed periodically with winds from the west. The gap in sampling starting in December 2018 was due to an extended U.S. Federal Government shutdown. The canister samples were periodically changed out approximately once every two weeks. For canisters that were not automatically triggered, representing 34 of 61 samples, the field person manually triggered or acquired the sample during the site visit under "as-encountered" conditions without regard to wind direction or SPod signal level. Automatic samples were triggered once a PID reading reached a threshold value of 4,000 cts to 7,000 cts, depending on the sensor type and setting. Approximately 4 automatically triggered canisters were caused by artifactual PID signal caused by rapid humidity and temperature changes and are similar in form to manually acquired samples as they represent conditions that do not correspond to emission source driven elevated PID readings. Three of the 61 canisters were 1 minutes in duration and were triggered by a prototype field gas chromatograph system (MiTAP® P310, Tricorntech Corp., Taipei City, Taiwan) that was collocated at the monitoring site for part of the larger study conducted at this

location[4]. Canister samples were screened for quality checks and were associated with a wind direction by linking the time of a grab sample to the collocated SPod readings during that time.

Figure S4: Distribution of TDLs

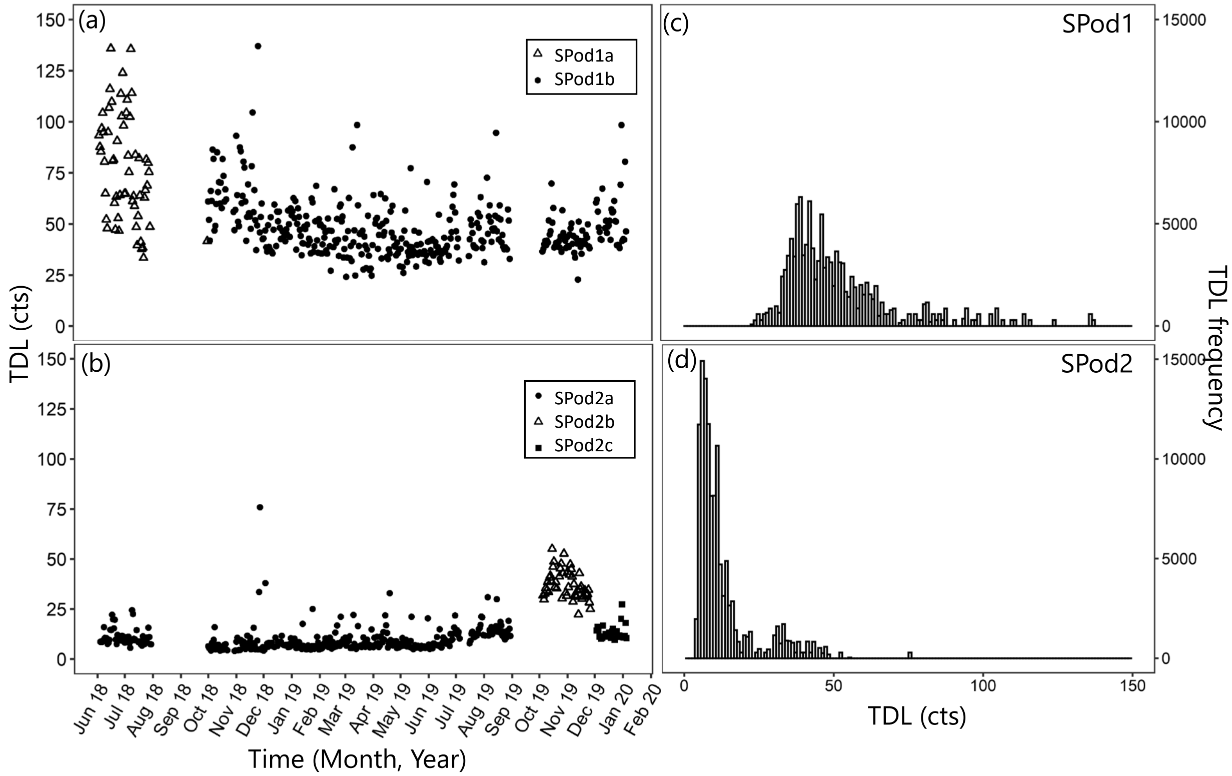

Figure S4: Daily background corrected TDL values for QA-valid paired data from units of (a) SPod1 and (b) SPod2. SI Figure 1(c) and 1(d) present distribution of combined TDLs for units of SPod 1 and SPod 2, respectively. The blank area between August 2018 and October 2018 was caused by failed PID in SPod1 while the blank area between September 2019 and October 2018 was caused by failed PID in SPod2. Three highest TDL values from SPod1 in (a, c): 425 cts on 11/27/18, 360 cts on 3/14/19, and 230 on 12/3/18 are not displayed (off scale high) for ease of viewing.

Daily background corrected TDL values, calculated as 3 times the median standard deviation of aggregated 5-minute data points, were higher for SPod1 than SPod2 due to differences in overall sensitivity and noise levels. The exception was SPod2b which exhibited elevated noise levels, but similar response sensitivity compared to other SPod2 sensors It is believed that the elevated noise level of SPod2b was caused by an issue with the prototype electronics of the SPod unit and not the PID sensor itself. Three high values are not shown in Figure S5 for ease of viewing. For example, the TDL of SPod1b on 11/27/18 of 425 counts. This day encompasses the highest TDL value seen in SPod2 as well. Elevated source signal plumes occurring throughout the day corresponded to a known (reported) issue at the facility to the west. This consistent signal caused the TDL value to be higher than other days in the study but was likely a result of elevated source signals and not elevated sensor noise. This represents a partial breakdown of the TDL calculation strategy since the lack of low signal time periods creates an artificially high TDL calculation, potentially eliminating some proportion of valid source signal. The second highest TDL level from SPod1 occurred on 3/14/19 when the sensor malfunctioned for a significant portion of the day due to environmental conditions (likely water on the sensor).

Figure S5: Long-Running Sensors and Detection Levels Overtime

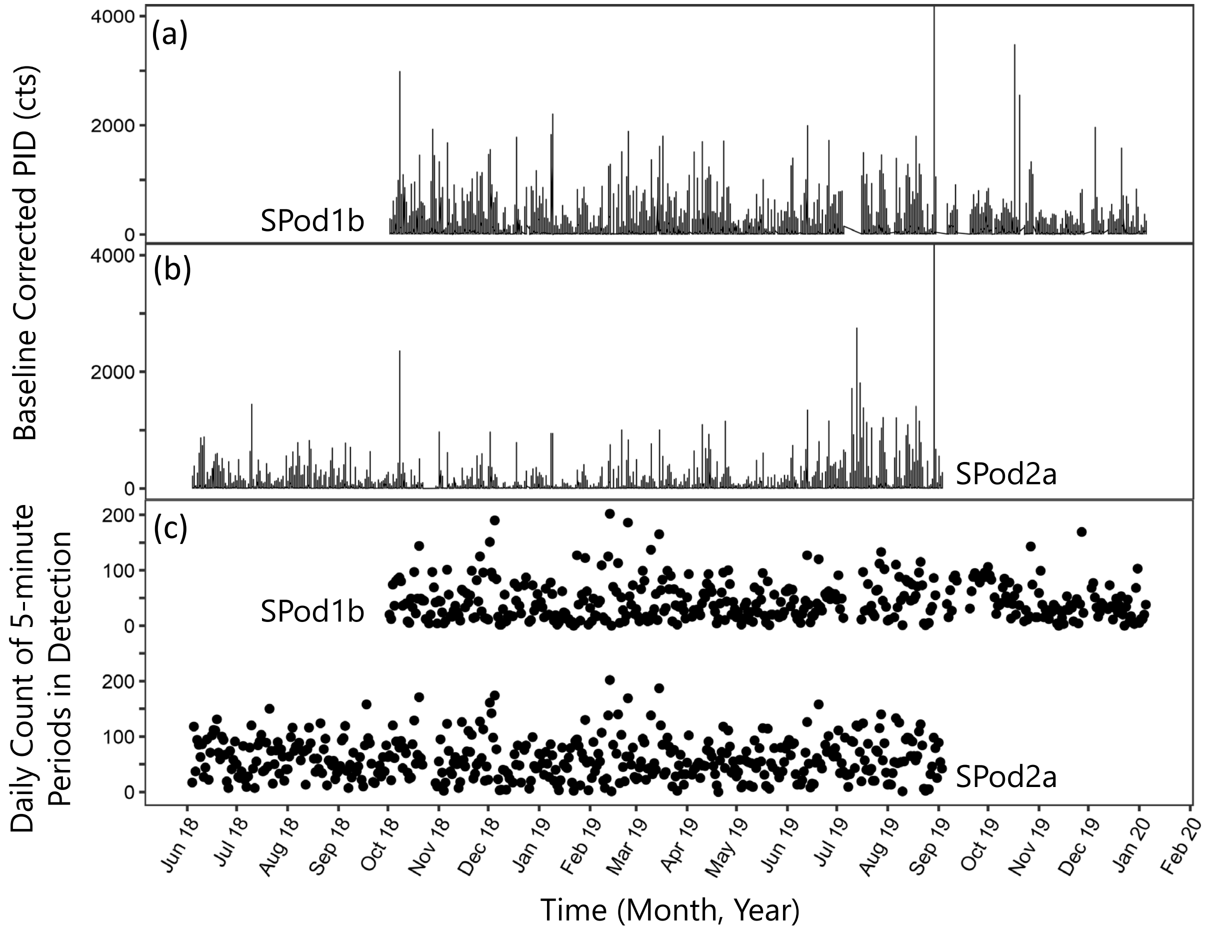

Figure S5: The QA-passing (not paired) datasets from the two longest-running sensors, SPod1b and SPod2a, compared by PID level after baseline correction algorithm (a-b) and sum of daily 5-minute periods in detection (above daily TDL) (c) over time. One high value continues out of graph range on 8/29/19 where PID level exceeded 8000 cts for SPod1b and 7000 cts for SPod2a.

Figure S5 summarizes the baseline-corrected PID data and the daily count of 5-minute periods greater than the TDL for the two longest-running sensors (SPod1b [10,094 hours] and SPod2a [10,778 hours]). We have found long-term deployments of PID sensor elements in this form typically lose 20% -30% sensitivity over the manufacturer's recommended 10,000 hours PID sensor lifetime [5]. Deterioration in senor element sensitivity can be exacerbated by harsh deployment conations and as discussed, sensors can fail prematurely. Assuming relatively stable VOC source loading over time, the data from this study imply that the heated PID sensors can operate in field conditions for extended periods of time without major loss in sensitivity or signal to noise performance. The highest PID values for both sensors were recorded on 8/29/19, where both sensors recorded 5-minute average PID values that exceeded were 7,000 cts after baseline correction. The period of overlap between these two long-running sensors is from October 2018 to September 2019, almost 1 year. From June 2018 to August 2018, SPod2a operated while being collocated with SPod1a and from October 2019 to January 2020 SPod1b operated while being collocated with SPod2b and SPod2c. While only the longest running sensors are depicted in Figure S5, the trend of sensor agreement continued between the sensors shown and SPod1a, SPod2b, and Spod2c.

Figure S6: Daily Distribution of PID values Above TDL

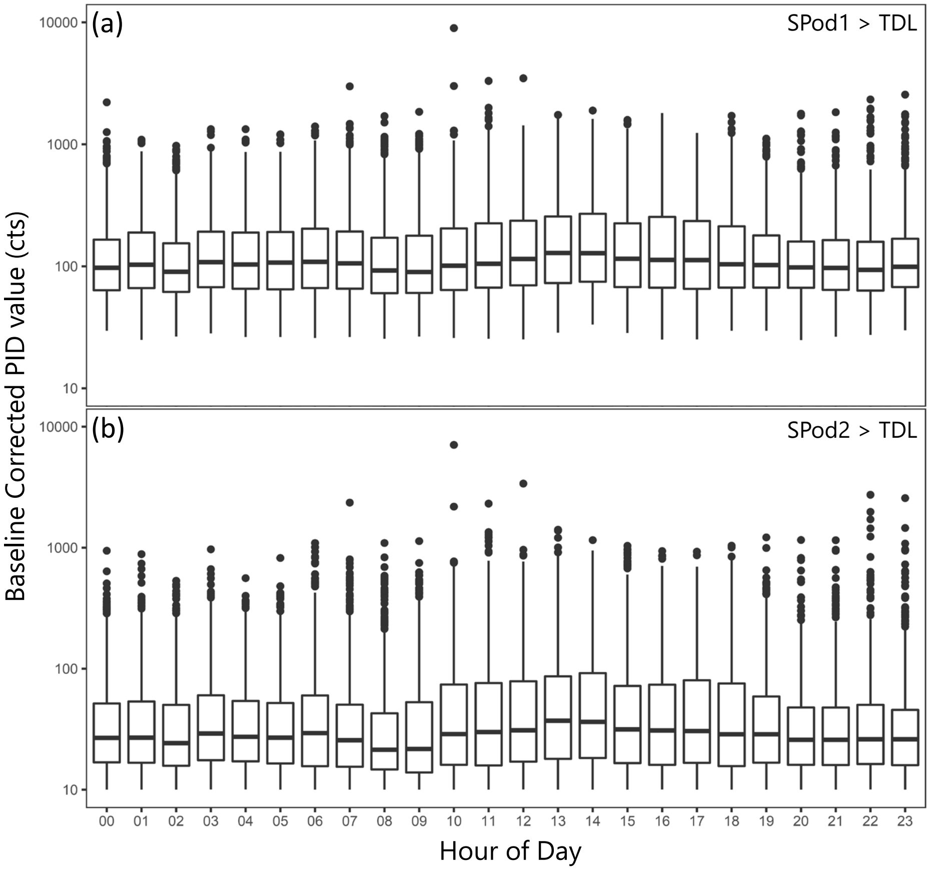

Figure S6: 24-hour boxplots of (a) SPod1 and (b) SPod2 above TDL datasets binned by hour. Outliers are depicted as black dots while the horizontal line indicates the mean value for each hour period. PID values are measured on a log scale in counts for ease of viewability.

SPod signal by hour of the day was similar over the course of the study. Although an analysis of the temporal distribution of the observed source emissions signal is beyond the scope of this paper, there was no obvious evidence of elevated concentrations during higher vehicle traffic time periods (rush hour). This supports the conclusion of little relative impact of vehicle traffic on the presented fenceline sensor signal. Whereas individual vehicles could in theory produce a brief signal on the SPods, the effect was insignificant in the five-minute averages as the traffic volume on the proximate roadway was low. This result is in part a consequence of the data processing used that removes slowly varying VOC air shed signals which will accumulate because of mobile sources and other source emission in the vicinity of the monitoring site during overnight calms. The conclusion of little vehicle impact on the PID sensor signal in this study should not be extrapolated to other sites or alternate fenceline sensor data analysis approaches.

Figure S7: Other Possible TDL Threshold Values

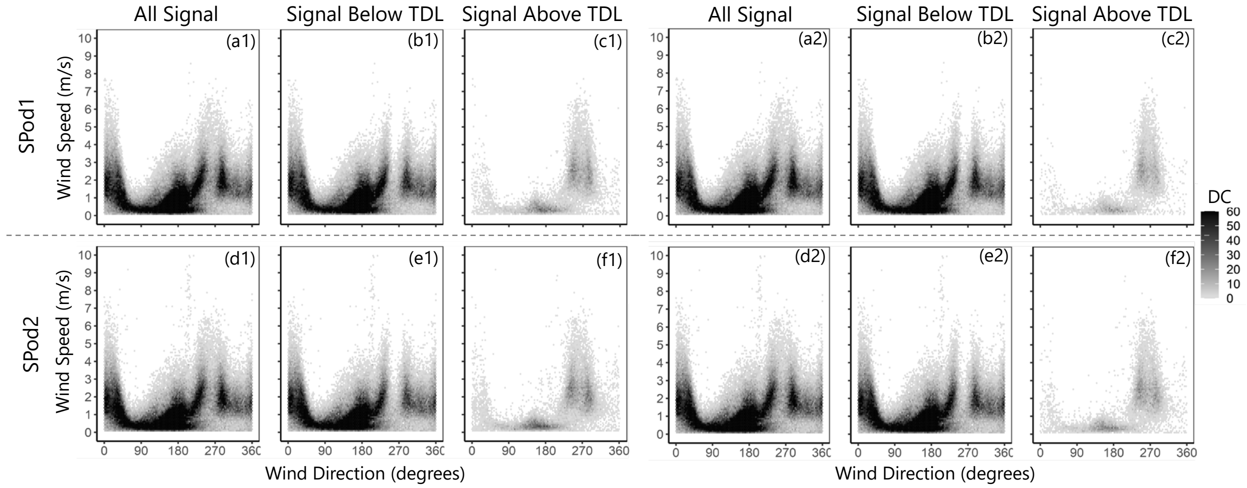

Data Summary (TDL = 6x)	SPod1	SPod2
Average [Median] of Daily TDL (cts)	107.0 [93.5]	24.8 [18.5]
Minimum [Maximum] of Daily TDL (cts)	46.0 [850.2]	8.2 [151.7]
Percentage of QA-valid Data > TDL (%)	10.2	13.5
QA-valid Paired Data Points > TDL (%)	10.0	13.4

Data Summary (TDL = 9x)	SPod1	SPod2
Average [Median] of Daily TDL (cts)	160.4 [140.3]	37.2 [27.8]
Minimum [Maximum] of Daily TDL (cts)	68.4 [1275.3]	12.3 [227.6]
Percentage of QA-valid Data > TDL (%)	6.5	9.1
QA-valid Paired Data Points > TDL (%)	6.3	9.0

Figure S7: DPC plots with the TDL set at 6 times the daily median standard deviation value for SPod1 [(a1),(b1),(c1)] and SPod2 [(d1),(e1),(f1)] and the TDL set at 9 times the daily median standard deviation value for SPod1 [(a2),(b2),(c2)] and SPod2 [(d2),(e2),(f2)],with (a1-2),(d1-2) including All Data, (b1-2),(e1-2), consisting of a subset of data Below the TDL, and (c1-2),(f1-2), consisting of a subset of data Above the TDL.

While this analysis utilized three times the daily median standard deviation of corrected PID values (3 x σ_i) as a TDL, it is also possible to utilize other multipliers. Figure S7 shows a multiplier of six times in the left panel and nine times in the right panel. These higher thresholds retain the same form of the data in the DC matrices, seen particularly at the area of above-TDL signal around 270 degrees. The amount of data remaining in the QA-valid, Above TDL subset shrinks to 6.3% for SPod1 and 9.0% for SPod2. We elected to set the TDL at three times the daily median standard deviation to prevent data loss while also maintaining the form of this data density matrix.

References

1. Brantley, H., J. Guinness, and E. Chi, *detrendr: Quantile Trend Filtering*, in *Baseline Drift Estimation for Air Quality Data using Quantile Trend Filtering*. 2020: https://arxiv.org/pdf/1904.10582.pdf.
2. MacDonald, M., et al. *Fenceline and Community Sensor Applications and Comparisons*. in *Air Sensors International Conference 2021*. https://www.youtube.com/watch?v=ACFm8-WhMRU.
3. Carslaw, D.C. and K. Ropkins, *Openair—an R package for air quality data analysis.* Environmental Modelling & Software, 2012. **27**: p. 52-61.
4. Thoma, E., et al., *Rubbertown Next Generation Emissions Measurement Demonstration Project.* International Journal of Environmental Research and Public Health, 2019. **16**(11): p. 2041.
5. *High Sensitivity VOC Gas Sensor*. Ion Science USA. 2022 [cited 2022 February 28]; Available from: https://ionscience.com/usa/products/minipid-2-hs-high-sensitivity-gas-sensor/#technical-specification.
